# Supplementary material for: Organic Salts of Pharmaceutical Impurity p-Aminophenol
Source: Molecules. 2020 Apr 21;25(8):1910. doi: 10.3390/molecules25081910 (PMC7221883; doi:10.3390/molecules25081910)
Supplement: Supplementary file 1 [file molecules-25-01910-s001.pdf]

Organic Salts of Pharmaceutical impurity *p*-Aminophenol

U. B. Rao Khandavilli, Leila Keshavarz, Eliška Skořepová, René R. E. Steendam, Patrick J. Frawley

**IR data**	**S2-S3**
**PXRD data**	**S4-S5**
**DSC data**	**S6-S7**
**TGA data**	**S8-S9**
**UV data**	**S10**
**PSD analysis**	**S10**
**SEM images**	**S11**
**HPLC data**	**S11-S12**
**Crystal images and dendrogram**	**S13**
**Reference**	**S14**

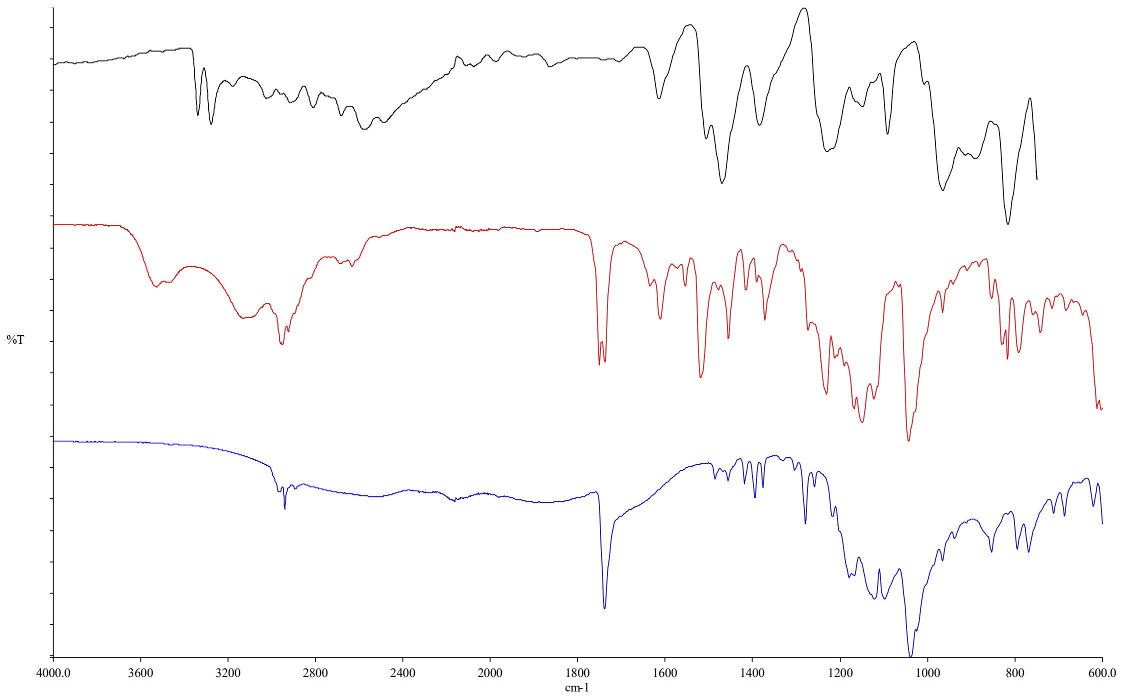

Figure S1. IR spectra comparison for PAP (black), (+)-Camphorsulphonic acid (red) and $PAP^{+}CSA^{-}$ (blue)

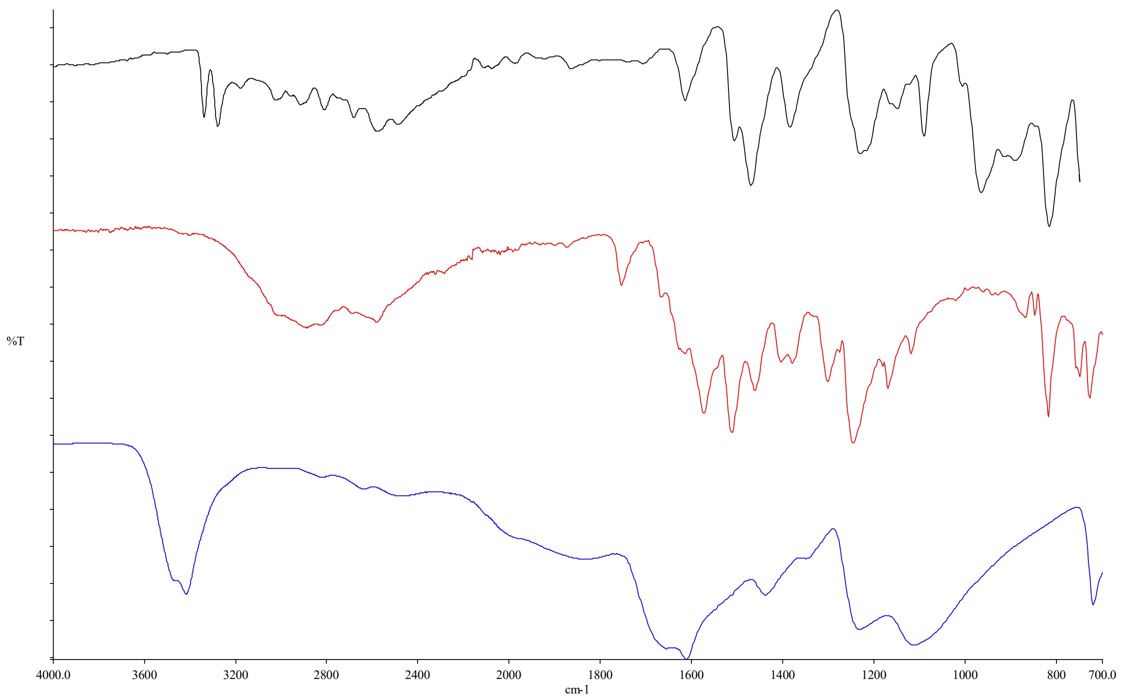

Figure S2. IR spectra comparison for PAP (black), Oxalic acid (red) and $PAP^{+}OX^{-}$(blue)

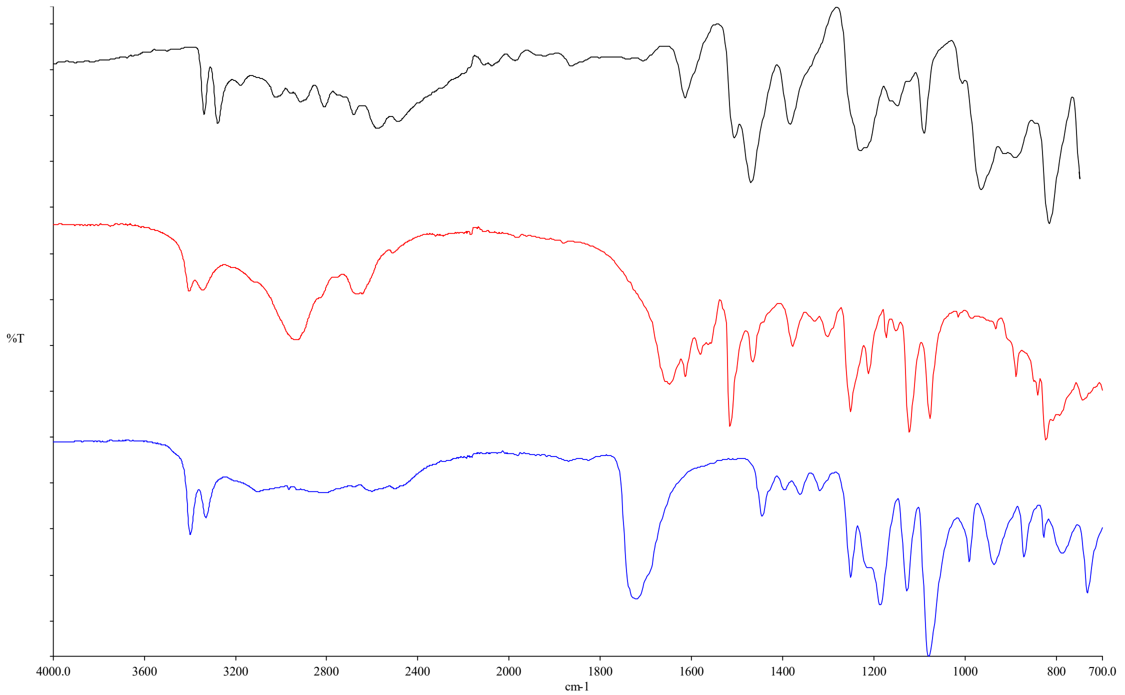

Figure S3. IR spectra comparison for PAP (black), (L)-Tartaric acid (red) and $PAP^{+}TA^{-}$ (blue)

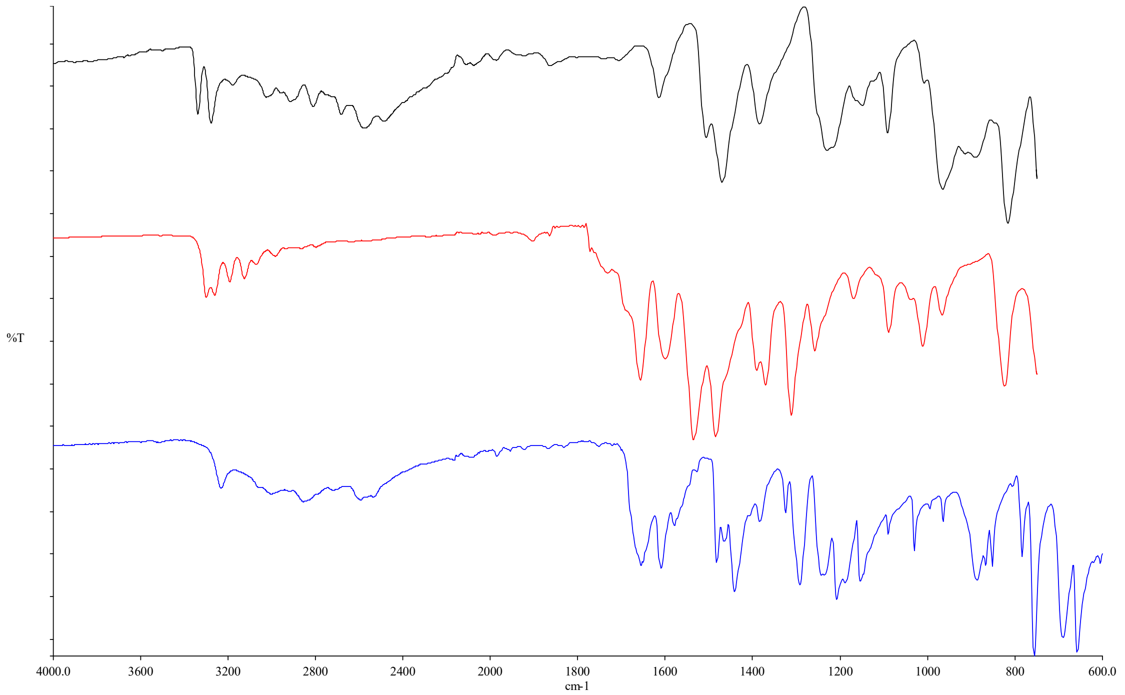

Figure S4. IR spectra comparison for PAP (black), Salicylic acid (red) and $PAP^{+}SA^{-}$ (blue)

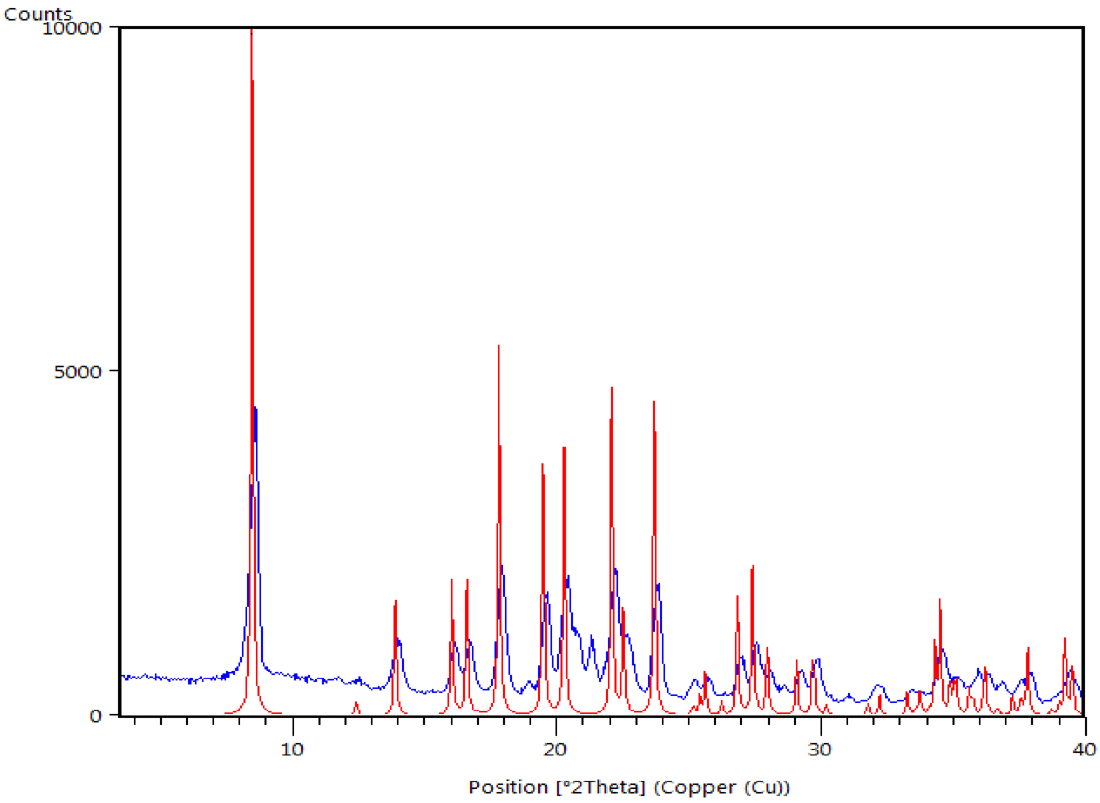

Figure S5. PXRD data for $PAP^{+}TA^{-}$: experimental (blue) and theoretical (red)

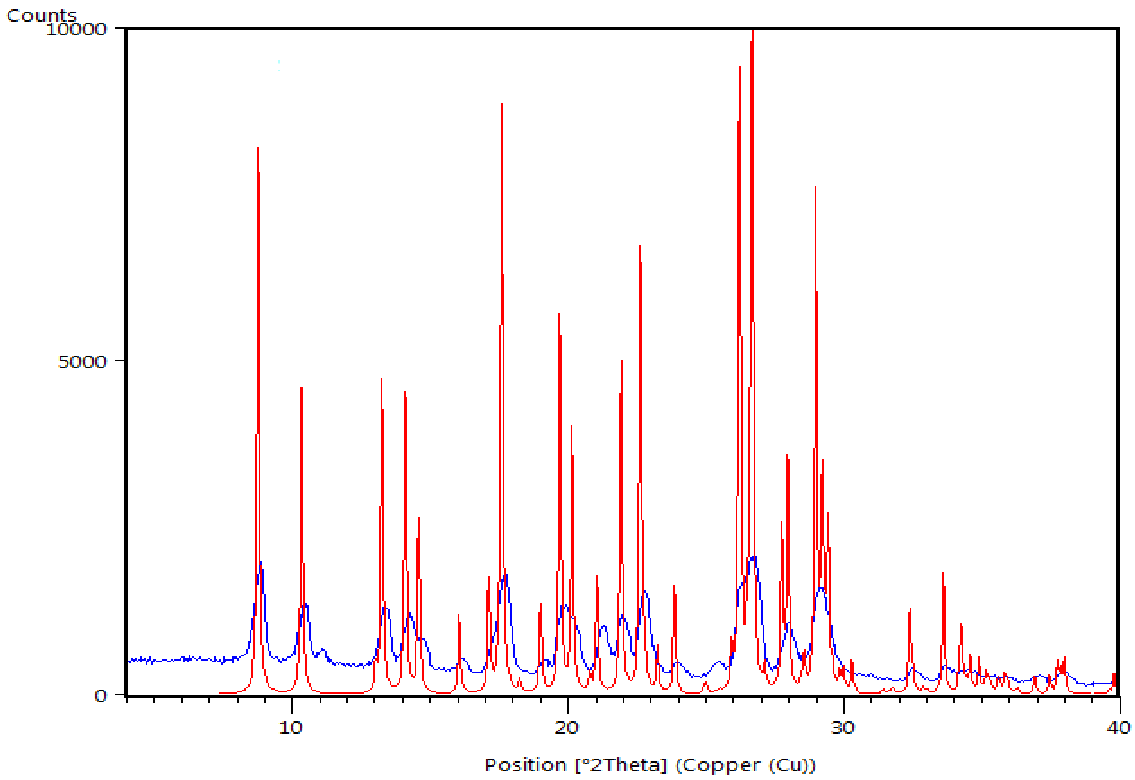

Figure S6. PXRD data for $PAP^{+}SA^{-}$: experimental (blue) and theoretical (red)

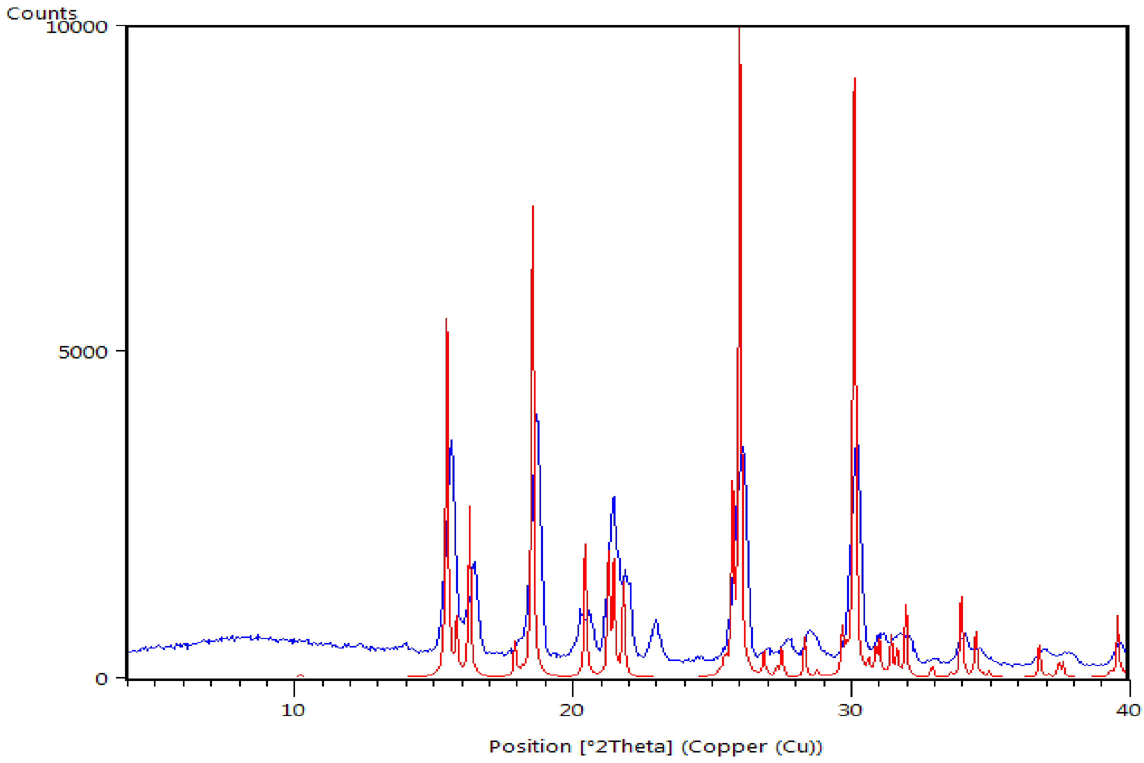

Figure S7. PXRD data for PAP$^+$OX$^-$: experimental (blue) and theoretical (red)

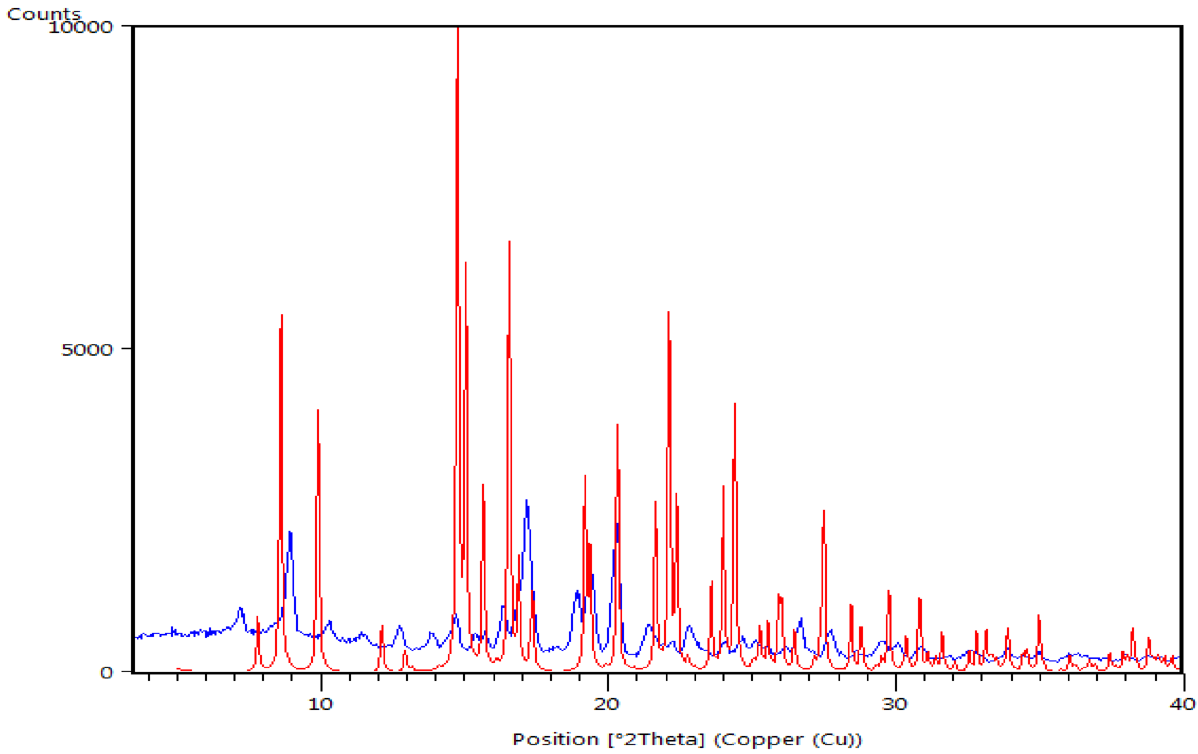

Figure S8. PXRD data for PAP$^+$CSA$^-$: experimental (blue) and theoretical (red)

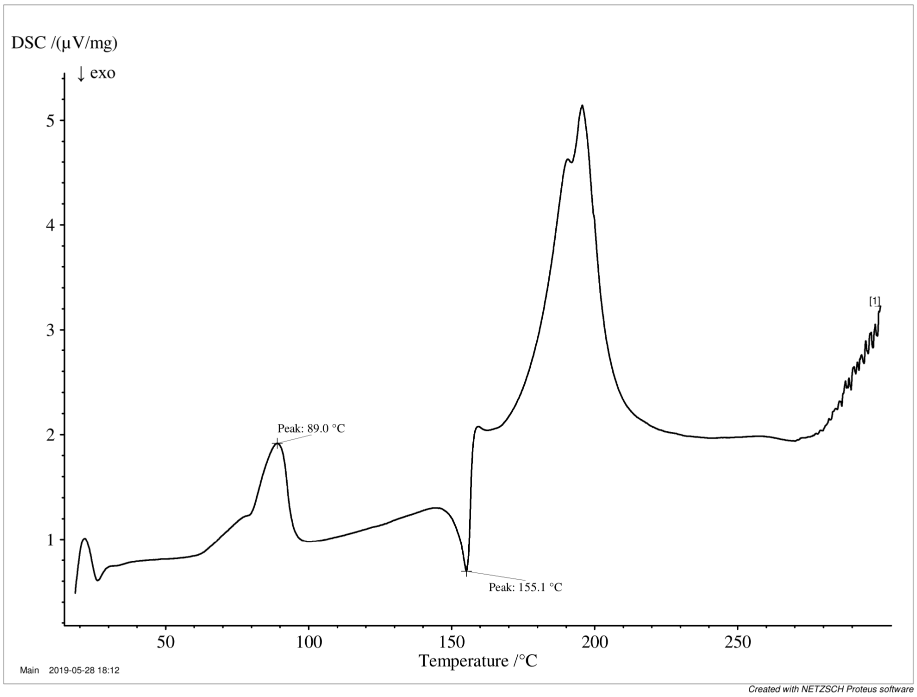

Figure S9. DSC data for $PAP^{+}CSA^{-}$

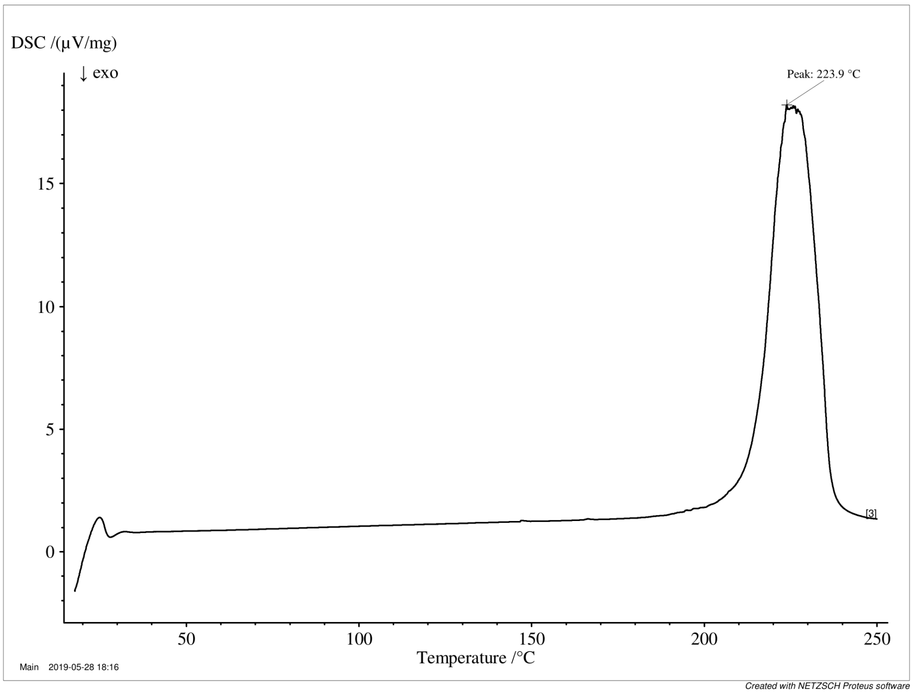

Figure S10. DSC data for $PAP^{+}TA^{-}$

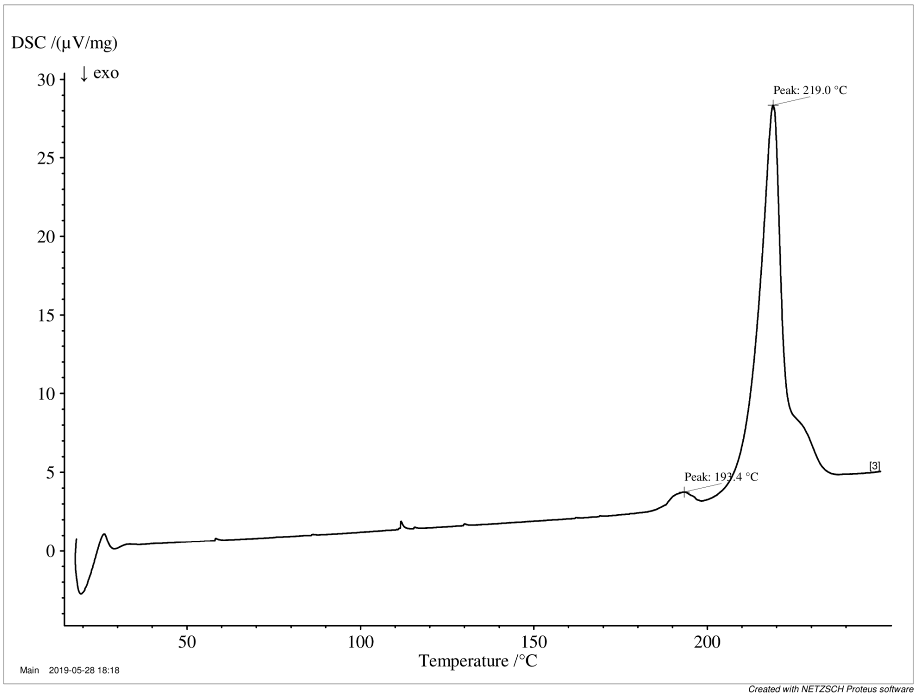

Figure S11. DSC data for $PAP^{+}OX^{-}$

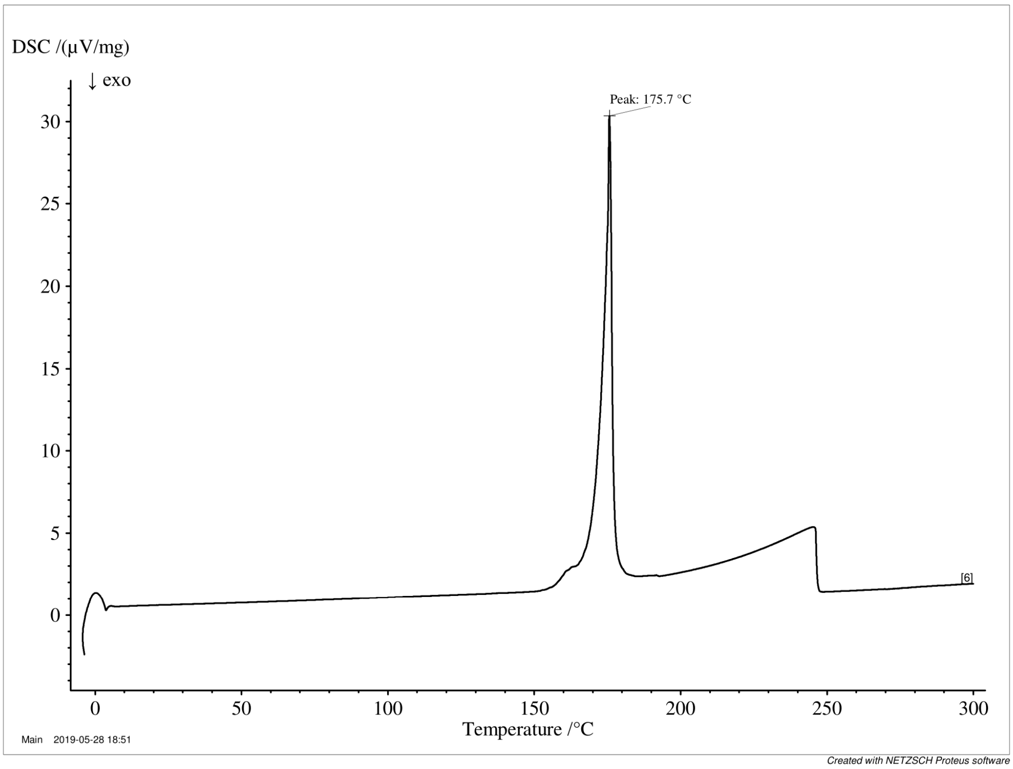

Figure S12. DSC data for $PAP^{+}SA^{-}$

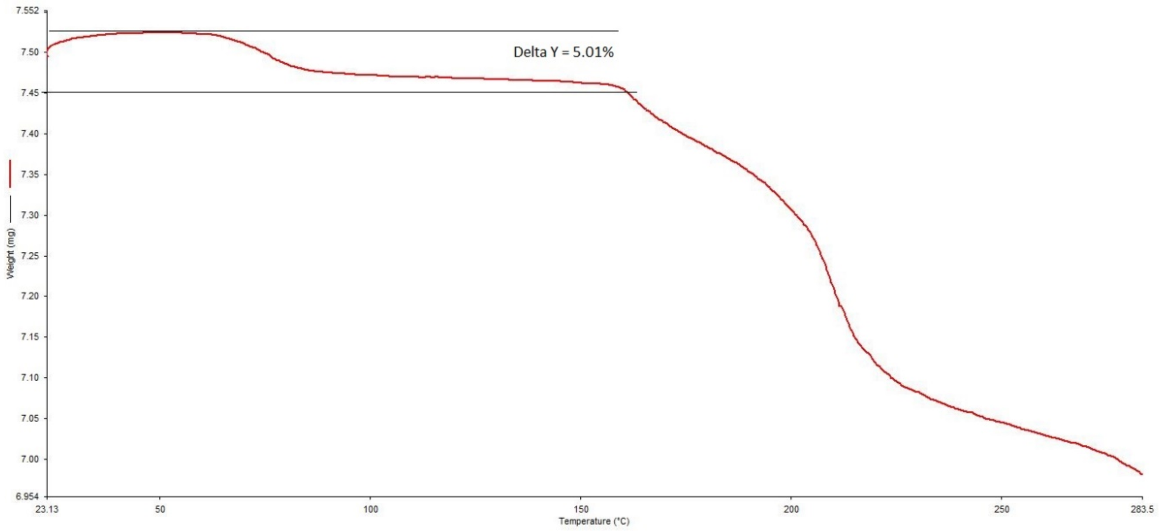

Figure S13. TGA data for PAP$^+$CSA$^-$

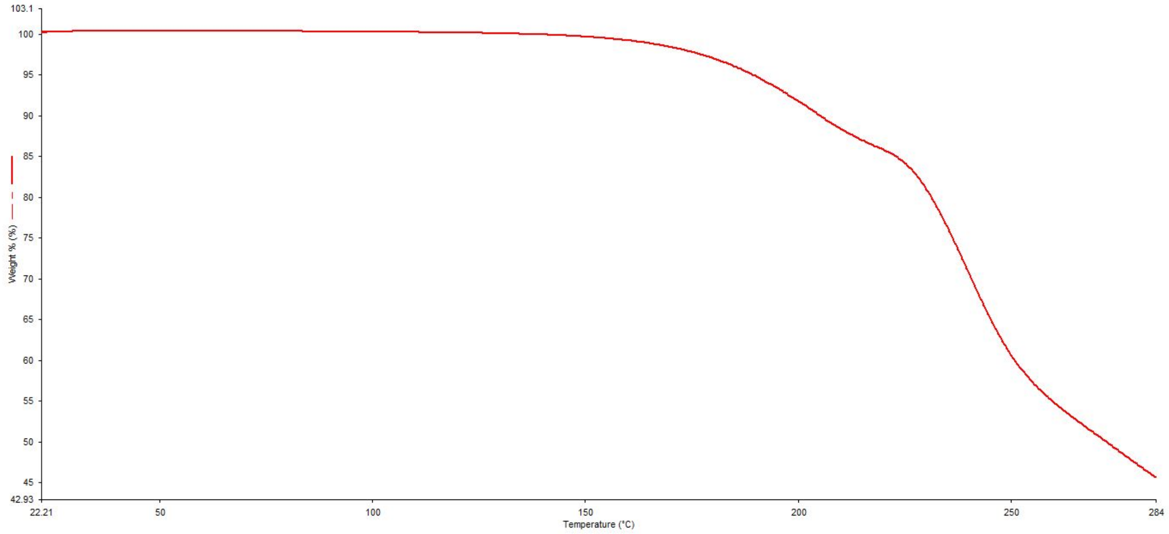

Figure S14. TGA data for PAP$^+$OX$^-$

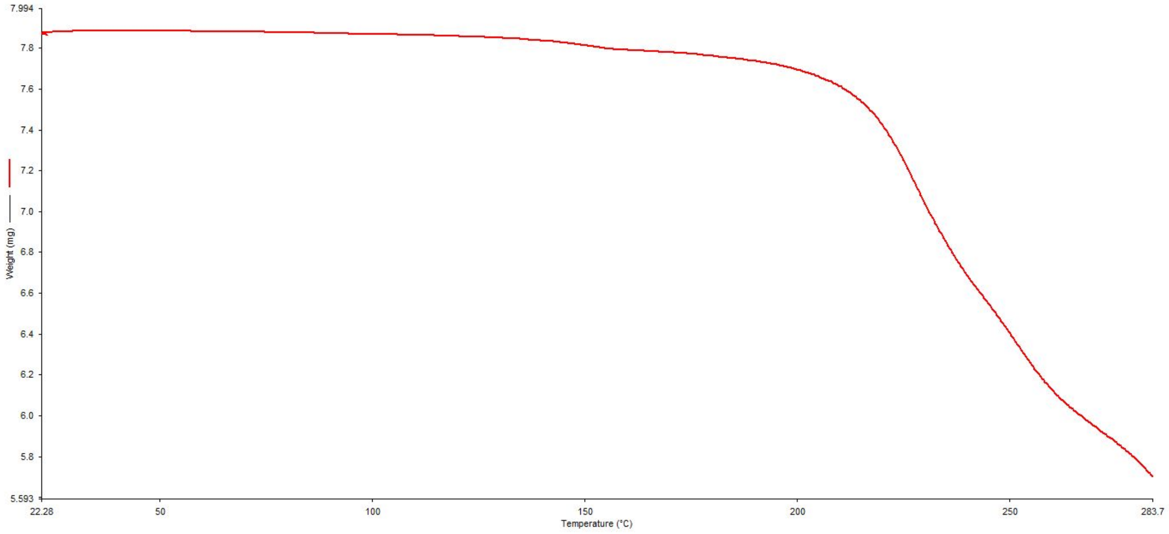

Figure S15. TGA data for $PAP^{+}TA^{-}$

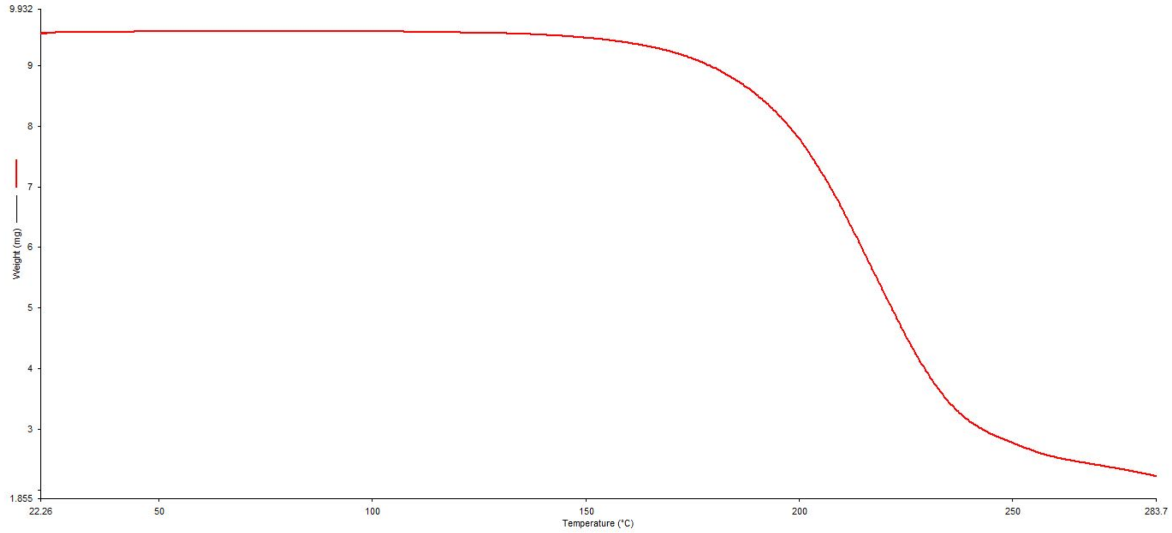

Figure S16. TGA data for $PAP^{+}SA^{-}$

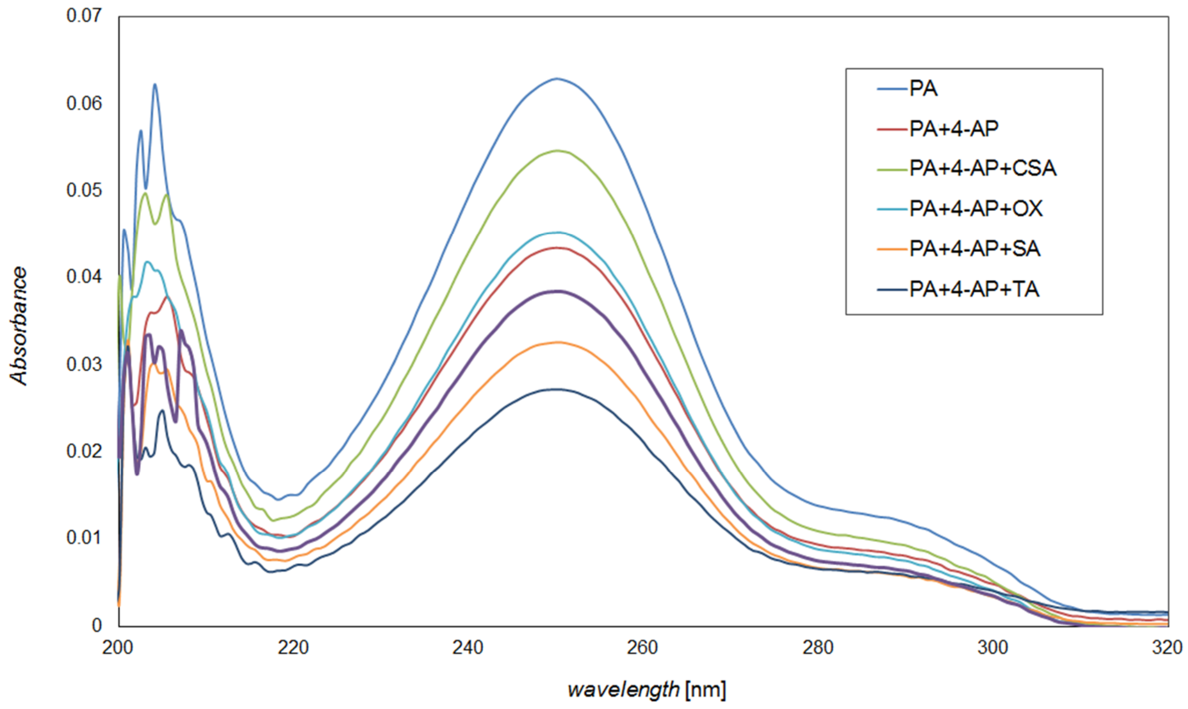

Figure S17. UV experiments on paracetamol obtained from the different experiments

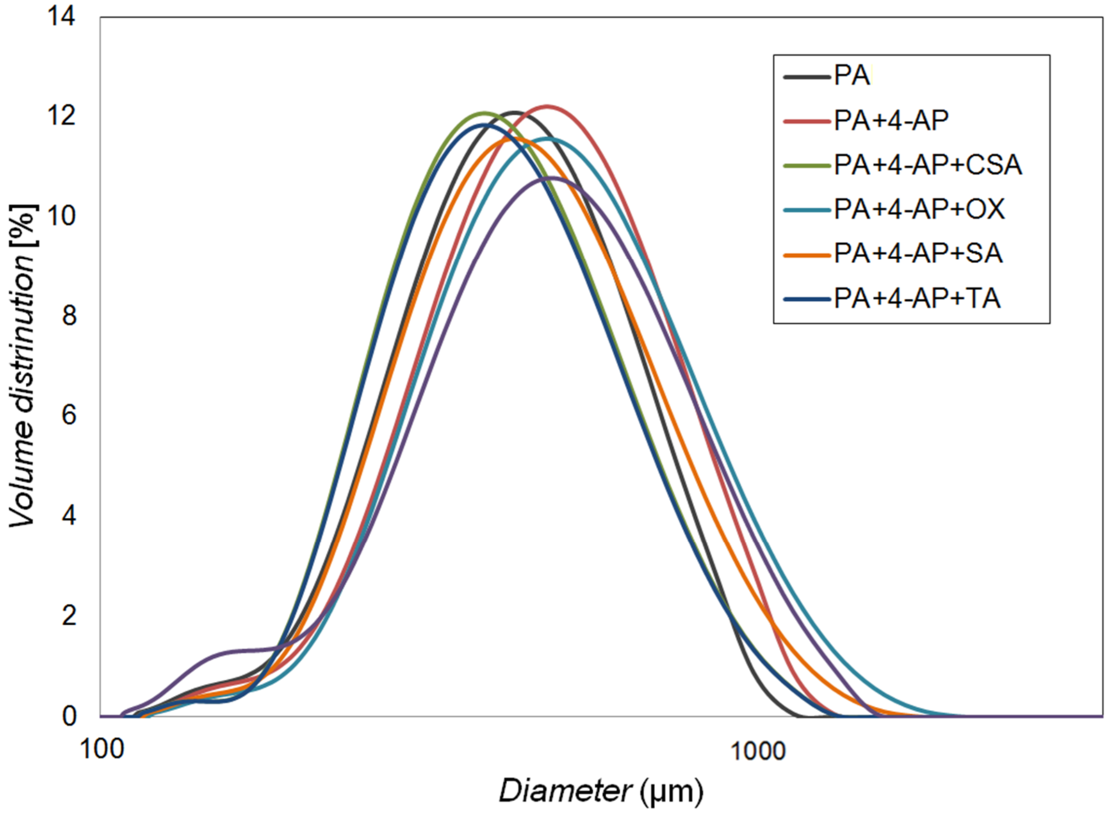

Figure S18. Particle size analysis on the paracetamol crystals obtained from the above experiments

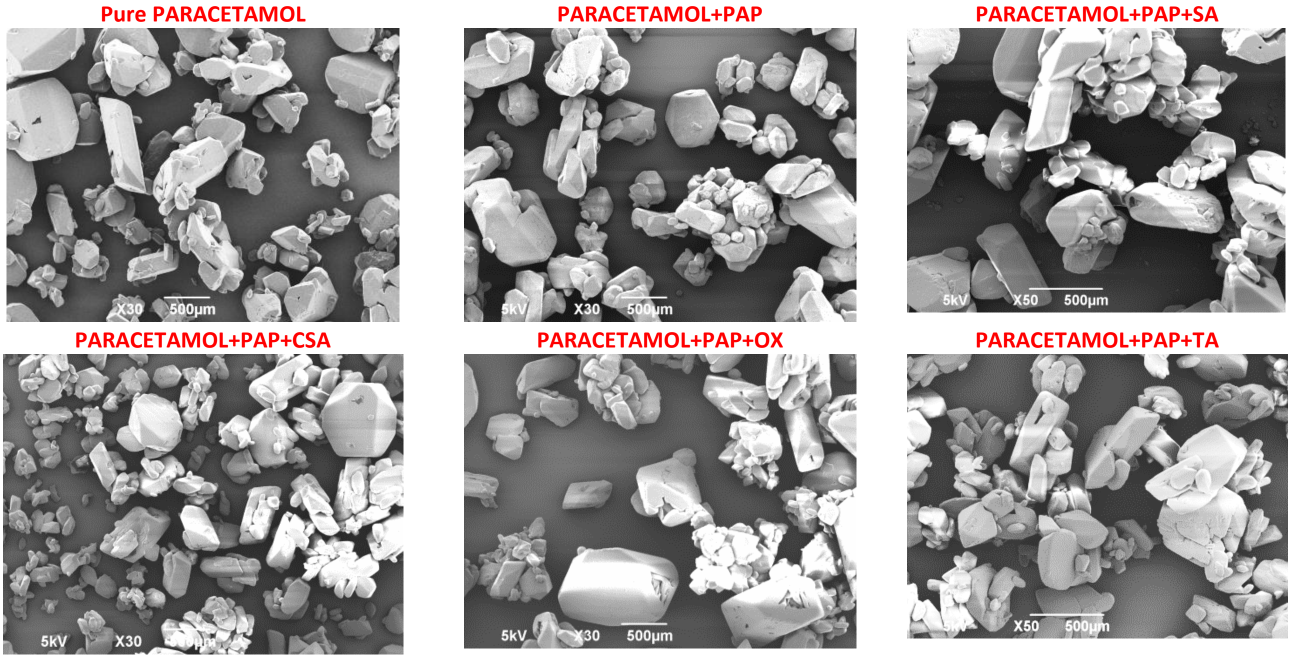

Figure S19. SEM images of different paracetamol crystals obtained from the above experiments

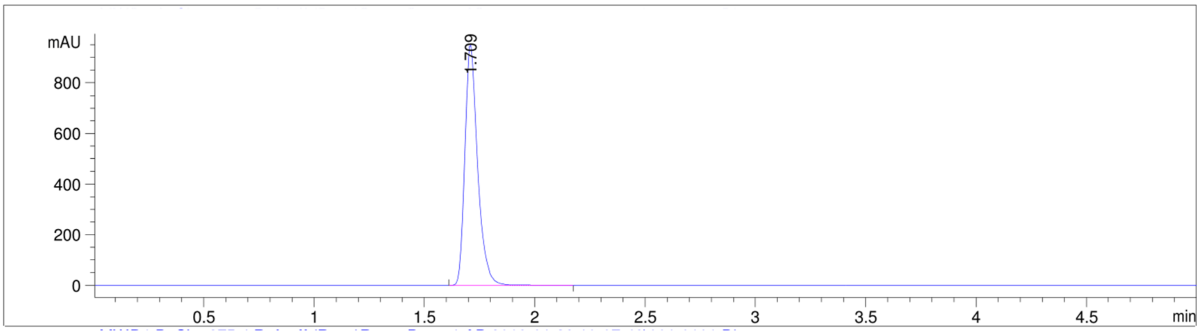

Figure S20. HPLC of Paracetamol pure

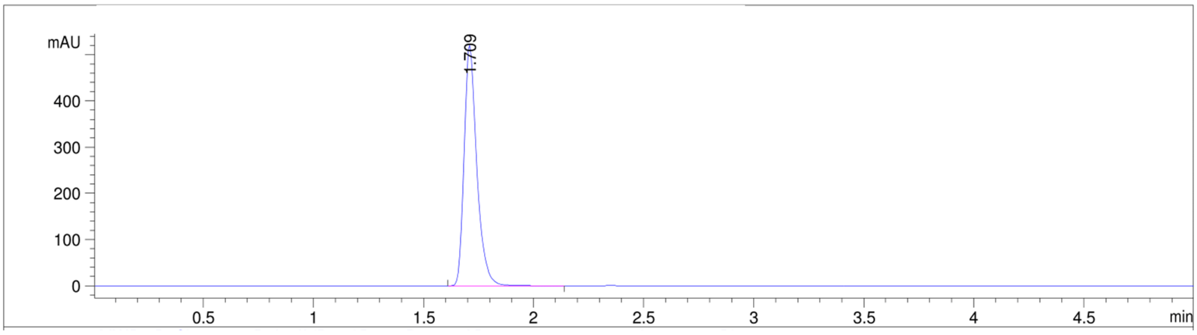

Figure S21. HPLC of Paracetamol spiked with PAP

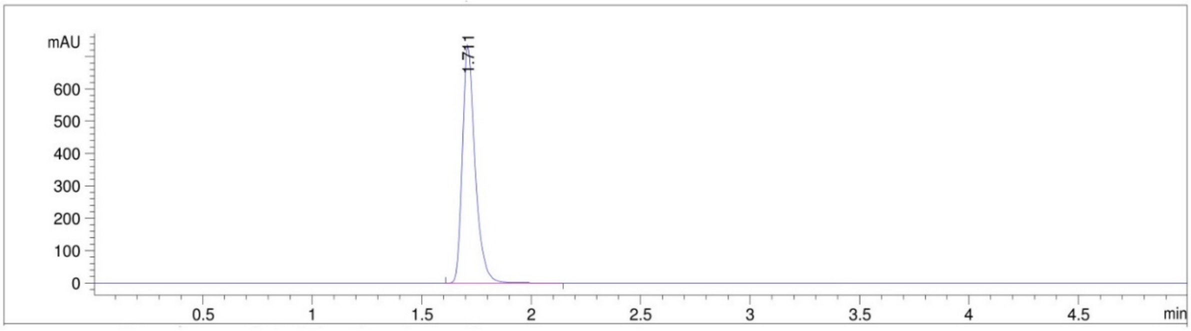

Figure S22. HPLC of Paracetamol spiked with PAP and CSA

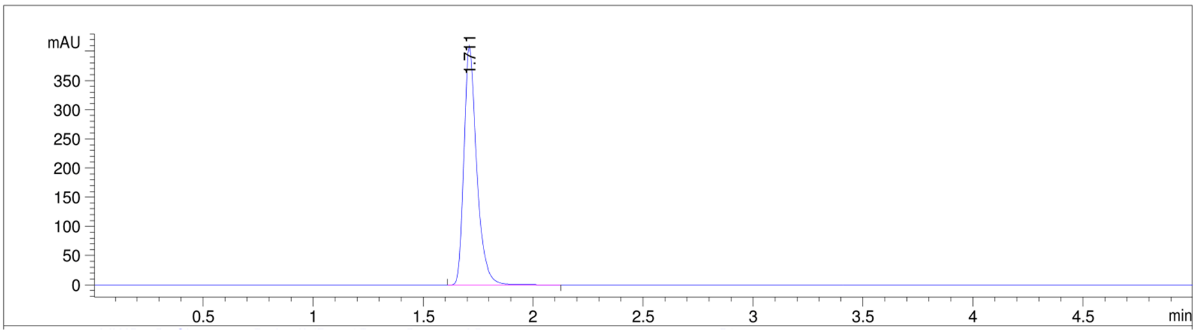

Figure S23. HPLC Paracetamol spiked with PAP and OX

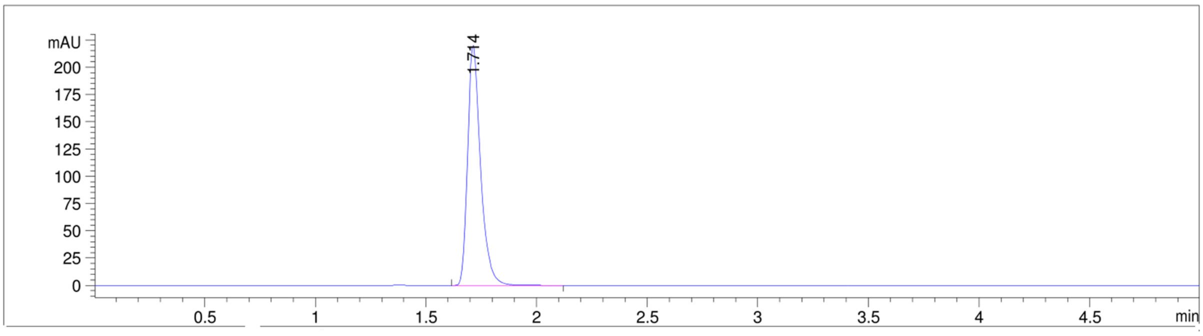

Figure S24. HPLC of Paracetamol spiked with PAP and SA

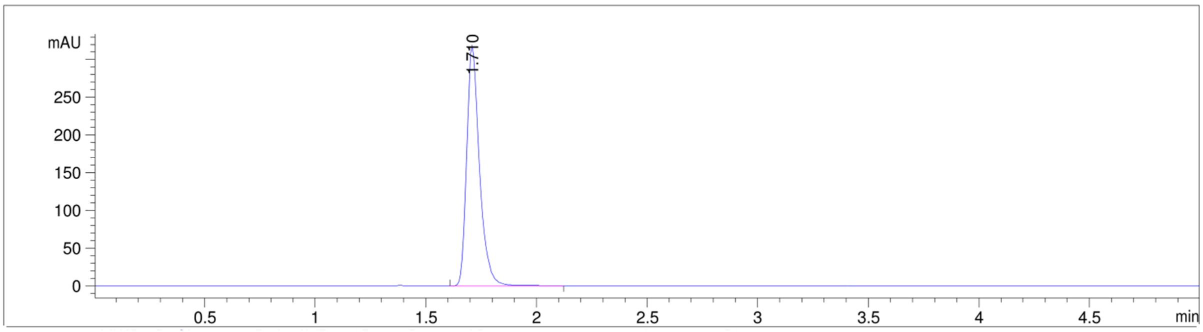

Figure S25. HPLC of Paracetamol spiked with PAP and TA

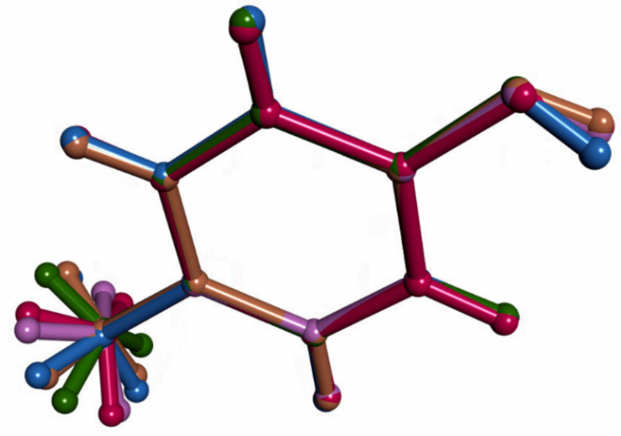

Figure S26. Overlay of the PAP cation conformations

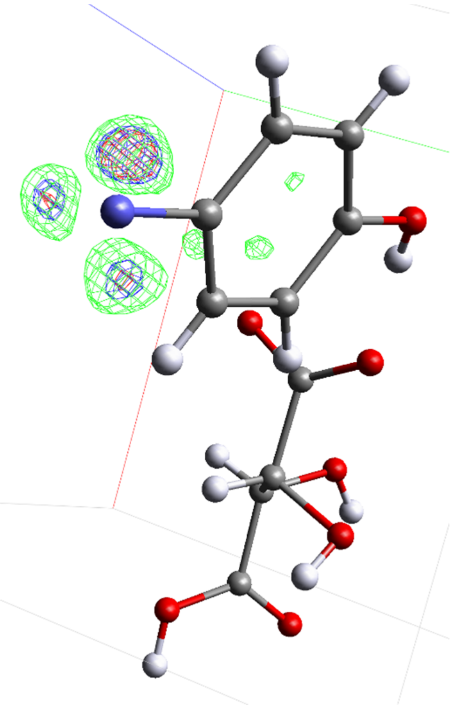

Figure S27. Difference electron density map of PAP TA salt shows the three hydrogen atom positions on the nitrogen.

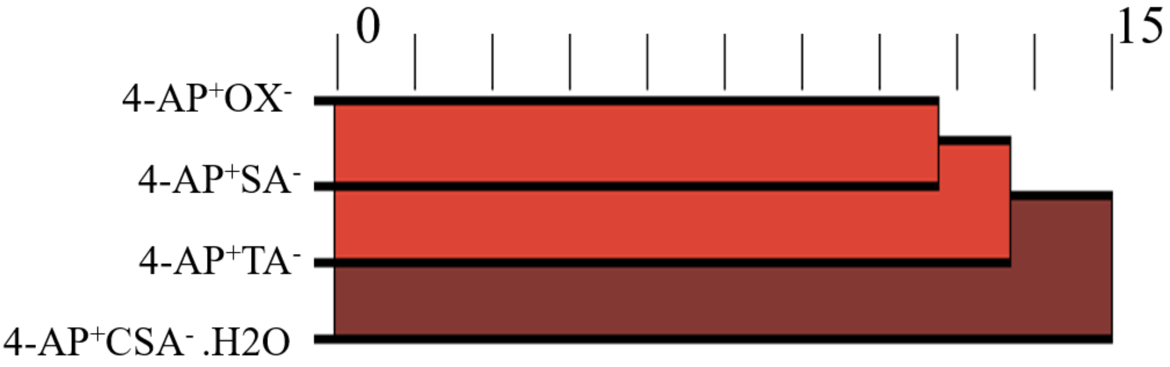

Figure S28. Packing similarity tree diagram (dendrogram) of PAP salts using CrystalCMP[1].

Reference:

1. Rohlíček, J.; Skořepová, E.; Babor, M.; Čejka, J. CrystalCMP: an easy-to-use tool for fast comparison of molecular packing J. Appl. Crystallogr. 2016, 49, 2172-2183.
